# Prospective Exploratory Study of the Clinical Significance of Circulating Tumor Cells in Patients With Small Cell Lung Cancer Exposed to Prophylactic Cranial Irradiation

**DOI:** 10.3389/fonc.2020.575394

**Published:** 2021-02-08

**Authors:** Lei Deng, Ye Zhang, Wen Zhang, Lin Feng, Kaitai Zhang, Wenqing Wang, Zongmei Zhou, Luhua Wang, Zhouguang Hui

**Affiliations:** ^1^ Department of Radiation Oncology, National Cancer Center/National Clinical Research Center for Cancer/Cancer Hospital, Chinese Academy of Medical Sciences and Peking Union Medical College, Beijing, China; ^2^ Department of Immunology, National Cancer Center/National Clinical Research Center for Cancer/Cancer Hospital, Chinese Academy of Medical Sciences and Peking Union Medical College, Beijing, China; ^3^ State Key Laboratory of Molecular Oncology, Department of Etiology and Carcinogenesis, National Cancer Center/National Clinical Research Center for Cancer/Cancer Hospital, Chinese Academy of Medical Sciences and Peking Union Medical College, Beijing, China; ^4^ Department of Radiation Oncology, National Cancer Center/National Clinical Research Center for Cancer/Cancer Hospital & Shenzhen Hospital, Chinese Academy of Medical Sciences and Peking Union Medical College, Shenzhen, China; ^5^ Department of VIP Medical Services, National Cancer Center/National Clinical Research Center for Cancer/Cancer Hospital, Chinese Academy of Medical Sciences and Peking Union Medical College, Beijing, China

**Keywords:** circulating tumor cells, small cell lung cancer, prophylactic cranial irradiation, radiotherapy, prognosis

## Abstract

**Objective:**

Circulating tumor cells (CTCs) can predict the efficacy of anti-cancer treatments and indicate prognosis. Here we investigate the significance of CTCs in relation to the prediction of treatment efficacy and prognosis in patients with small cell lung cancer (SCLC) who have received prophylactic cranial irradiation (PCI).

**Methods:**

CTCs were detected in 20 patients with SCLC before and after PCI using the oHSV1-hTERT-GFP method. The primary endpoints were progression-free survival (PFS) and overall survival (OS).

**Results:**

Eleven patients had limited-stage SCLC, and nine had extensive-stage SCLC. All patients completed chemo-radiotherapy and received PCI. The median baseline CTC count before PCI was 12. After PCI, the median CTC count was 4. The median follow-up time for all enrolled patients was 39.2 months. The median PFS and OS were significantly reduced in patients with ≥4 CTCs after PCI compared to those with <4 CTCs (PFS, 28.1 months vs. not reached, p = 0.001; OS, not reached vs. not reached, p = 0.029). Seven of the 10 patients with ≥4 CTCs after PCI failed after treatment, whereas the10 patients with <4 CTCs after PCI remained alive without tumors. The median PFS and OS were significantly improved in patients who exhibited a rate of CTC decline of ≥58% after PCI compared with patients who exhibited a decline rate of <58% (PFS, 26.4 months vs. not reached, p = 0.006; OS, not reached vs. not reached, p = 0.029).

**Conclusion:**

In SCLC patients who receive PCI, the CTC count and rate of CTC decline after PCI significantly correlate with prognosis.

## Background

Small cell lung cancer (SCLC) accounts for 10% to 15% of all lung cancers, with the majority presenting at a stage of extensive disease and poor prognosis (1). According to the National Comprehensive Cancer Network (NCCN) guidelines, prophylactic cranial irradiation (PCI) is recommended for patients with limited-stage SCLC who respond well to chemo-radiotherapy and for patients with extensive-stage SCLC who have received effective chemotherapy treatment. PCI reduces the 3-year cumulative incidence of brain metastasis and increases the 3-year overall survival (OS) rate (2, 3). However, a recent study from Japan found that PCI reduced the incidence of brain metastasis but failed to improve the OS in patients with extensive-stage SCLC who underwent effective chemotherapy (4). Internationally, there has been some controversy over whether SCLC patients who are effectively treated with chemo-radiotherapy should undergo active follow-up consisting of magnetic resonance imaging (MRI) or PCI (5, 6). Therefore, the identification of molecular markers that can be used for early prediction of therapeutic efficacy and to guide individualized treatment of SCLC represents an important research direction.

Circulating tumor cells (CTCs) refer to tumor cells in the peripheral circulation (7). A large number of studies have shown that CTCs can predict the efficacy of anti-cancer treatment and indicate prognosis (8); however, for SCLC, studies have mainly focused on correlations among the pre- and post-chemotherapy CTC numbers and the prognosis or chemo-sensitivity of SCLC (9–18). To date, the role of CTCs in predicting therapeutic efficacy and evaluating prognosis in SCLC patients who have undergone PCI after effective chemo-radiotherapy treatment has not been reported.

We previously employed the human telomerase reverse transcriptase (hTERT) promoter-regulated oncolytic herpes simplex virus-1 (oHSV1-hTERT-GFP, independent intellectual property rights acquired) method to detect CTCs (19–21). This CTC detection method is based on previous findings, which show telomerase is in an activated state in more than 90% of tumors (22) and tumor cells display a high level of telomerase transcription. The oHSV1-hTERT-GFP technique possesses several advantages over the current CellSearch technology that is commonly used in China and other countries including high sensitivity, ease of use, and the ability to isolate and observe the morphology of CTCs (19, 20).

The present study aimed to use the oHSV1-hTERT-GFP technique to detect CTC numbers in chemo-radiotherapy-responsive SCLC patients before and after PCI. We then preliminarily explored the relationships between CTC numbers and prognosis in SCLC patients.

## Materials and Methods

### Inclusion and Exclusion Criteria

This study included patients with SCLC who received PCI in our hospital between September 2014 and February 2015. SCLC was staged according to the Veterans Administration Lung Study Group (VALSG) staging system and the staging system presented in the 7th edition of the AJCC (American Joint Committee on Cancer) Cancer Staging Manual. According to VALSG staging system, limited-stage disease is disease confined to the ipsilateral hemithorax, which can be safely encompassed within a radiation field; and extensive-stage disease is disease beyond the ipsilateral hemithorax, including malignant pleural or pericardial effusion or hematogenous metastases. Contralateral mediastinal and ipsilateral supraclavicular lymphadenopathy are generally classified as limited-stage disease. The detailed inclusion criteria were as follows: (1) a definitive pathological diagnosis of SCLC; (2) had received a computed tomography (CT) scan of the neck, chest and abdomen, an MRI of the brain, and a bone scan; (3) had a Karnofsky performance status (KPS) score >70 points and were >18 years old; (4) had only received first-line treatment; (5) were willing to undergo PCI; and (6) had signed informed consent documents. Patients were excluded from the study if they: (1) experienced progressive disease during primary chemo-radiotherapy treatment; (2) had a second primary tumor; (3) did not undergo PCI or did not complete the treatment; or (4) had incomplete follow-up data. This retrospective study was approved by the Ethics Committee, National Cancer Center/National Clinical Research Center for Cancer/Cancer Hospital, Chinese Academy of Medical Sciences and Peking Union Medical College (approval no. NCC2015 G-77). All patients provided written informed consent prior to treatment, and all information was anonymized prior to analysis.

### Treatment and Evaluation

All patients with limited-stage SCLC received four to six cycles of a cisplatin/carboplatin-based first-line chemotherapy regimen. After zero to two cycles of chemotherapy, the patients were administered with concurrent thoracic radiotherapy. All patients with extensive-stage SCLC received more than four cycles of a cisplatin/carboplatin-based first-line systemic chemotherapy. During chemotherapy, the patients were given standard antiemetics, hydration therapy, and other supportive/symptomatic treatments.

After every two cycles of chemotherapy, the tumors were examined by cervical and thoracoabdominal CT and brain MRI. The objective response of a tumor was evaluated according to the Response Evaluation Criteria in Solid Tumors (RECIST), version 1.1. Treatment was deemed effective if the tumor showed complete response (CR) or partial response (PR). All patients with extensive-stage SCLC who showed a PR to chemotherapy underwent thoracic radiotherapy. The target volume for thoracic radiotherapy was defined as follows: (1) gross tumor volume (GTV) included the primary lung lesions or the residual primary lesions after chemotherapy, as well as the metastatic mediastinal and supraclavicular lymph nodes or the remaining metastatic lymph nodes after chemotherapy; (2) clinical tumor volume (CTV) was defined as the primary lesion plus an additional 8-mm margin, as well as the affected mediastinal and supraclavicular lymphatic drainage areas before chemotherapy; (3) planning target volume (PTV) was generated by adding a 5-mm margin in all directions to the CTV. The prescribed radiation dose for 95% of the PTV volume was 60 Gy at 2 Gy in 30 fractions or 45 Gy at 3 Gy in 15 fractions. All radiotherapy regimens employed intensity-modulated radiation therapy (IMRT) techniques.

One month after the completion of chemotherapy and thoracic radiotherapy, treatment-responsive patients who showed no signs of brain metastasis were given PCI. The target volume was the whole brain. The prescribed dose was 25 Gy (2.5 Gy in 10 fractions). Radiation was provided two-dimensionally, by two parallel opposed fields.

All patients attended regular follow-up appointments after they completed the treatments. The patients were reexamined once every 3 months during the first 2 years and once every 6 months from the 3^rd^ to the 5^th^ year. The follow-up exams included CT of the neck, chest and abdomen, and MRI of the brain. Patients who exhibited an increased level of alkaline phosphatase, those who experienced whole-body aches or had fixed tender points were subjected to bone scans.

### Sample Collection and CTC Determination

Venous blood samples (4 ml each) were collected from all enrolled patients under fasting conditions prior to and after PCI (i.e., a total of two time points) and placed in blood collection tubes containing the anticoagulant EDTA (BD Biosciences, USA). Within 1 h of collection, the blood samples were transferred to the laboratory and subjected to the CTC test. For all patients, we divided the initial samples into two parts and tested them separately to get the results. The final result is the average count value of the two tests. The primary experimental steps were as follows (19):

1. The anticoagulated blood was incubated with red blood cell (RBC) lysis buffer (Qiagen, USA) to lyse the RBCs. The nucleated cells were isolated, resuspended in 2 ml of Roswell Park Memorial Institute (RPMI) 1640 medium (HyClone, USA), seeded into 6-well plates and labeled. Each well of the six-well plates was filled with 1 ml of single-cell suspension (4 ml of blood contained approximately 1 × 10^7^ nucleated cells). Subsequently, the oHSV1-hTERT-GFP virus was added to the wells containing the cell suspension (multiplicity of infection [MOI] = 1). After incubation in a humidified incubator at 37°C and 5% CO2 for 1 h, the cells were overlaid with 1 ml of complete medium containing 10% fetal bovine serum (FBS) and further incubated in the same incubator. The cells were harvested 20 to 24 h after viral infection; since the plaque-forming unit (PFU) for the virus was approximately 1.3 × 10^8^, 50 μl of virus was added to each well.
2. The cells were collected, and the leukocytes were labeled with anti-CD45 monoclonal antibody (HI30)-PECy5 (BD Biosciences, USA). The CTCs in the blood were isolated using a negative selection technique, as follows: the above cells were mixed with 400 μl of phosphate-buffered saline (PBS) and 100 μl of anti-CD45 antibody (in a 100-μl reaction system, 20 μl of CD45 antibody interacted with 1 × 10^6^ cells; each centrifuge tube contained approximately 5 × 10^6^ cells) and were then incubated for 30 min at room temperature (RT) in the dark. After incubation, each tube of cells was washed with 2 ml of PBS and centrifuged at 500 g for 5 min. The PBS was then discarded.
3. Examination of CTCs by flow cytometry (Guava, Millipore, USA) was performed as follows: single-cell suspensions were prepared and then added to 96-well plates at a density of 200 μl/well. After flow cytometric analysis, the data were recorded, processed and analyzed (the CD45-/GFP+ cells were defined as CTCs).

### Statistical Analysis

All statistical analyses were performed using SPSS 21.0 software. Logistic regression was employed to analyze the relationships among CTC counts and clinical variables. The rate of CTC decline after PCI was defined as: (the number of CTCs before PCI - the number of CTCs after PCI)/the number of CTCs before PCI × 100%. OS was defined as the time elapsed from the date of pathological diagnosis to the date of patient death, the date when the patient became lost to follow-up or the date of the last follow-up. Disease progression was defined as local or distant progression, patient death or loss to follow-up. Survival curves were plotted using the Kaplan-Meier method. The log-rank test was performed to compare the survival times between the two groups. A p-value less than 0.05 was considered statistically significant.

## Results

### Clinical Characteristics of the Patients

From September 2014 to February 2015, 20 patients with SCLC were enrolled in the present study. The clinical characteristics and treatments of the enrolled patients are summarized in **Table 1**.

**Table 1:** Baseline and clinical characteristics of the patients.

Clinical factors	Number of patients (%)		Clinical factors	Number of patients (%)
Gender			AJCC N stage (7^th^ edition)	
Male	13 (65%)		N1	3 (15%)
Female	7 (35%)		N2	8 (40%)
Age			N3	9 (45%)
Median (range)	60.5 (39–68)		AJCC stage (7^th^ edition)	
Smoking history			Stage IIb	2 (10%)
No smoking history	8 (35%)		Stage IIIa	5 (25%)
Former smoker	2 (10%)		Stage IIIb	6 (30%)
Current smoker	10 (50%)		Stage IV	7 (35%)
Smoking index (pack year)			VALSG stage	
Median (range)	20 (0–120)		Limited stage	9 (45%)
Primary site			Extensive stage	11 (55%)
Right upper lobe	2 (10%)		Chemotherapy cycle	
Right middle lobe	5 (25%)		Median (range)	6 (4–8)
Right lower lobe	7 (35%)		Chemotherapy efficacy	
Left upper lobe	4 (20%)		CR	4 (20%)
Left lower lobe	2 (10%)		PR	16 (80%)
AJCC T stage (7^th^ edition)			Thoracic radiotherapy	
T2	8 (40%)		60 Gy in 30 fractions	17 (85%)
T3	4 (20%)		45 Gy in 15 fractions	3 (15%)
T4	8 (40%)			

AJCC, American Joint Committee on Cancer; VALSG, Veterans Administration Lung Study Group; CR, complete response; PR, partial response.

The follow-up ended in January 2018. The median follow-up time for all enrolled patients was 39.2 months (range, 15.7–46.0 months), while the follow-up duration for those who survived was 40.0 months (range, 34.9–46.0 months). Seven patients failed, among whom five developed distant metastasis, one experienced local recurrence with lung metastasis, and one had mediastinal lymph node recurrence with liver metastasis. Of the five patients with distant metastasis, three had lung metastases, and two had brain metastases. Four patients died and all of them developed distant metastasis. Finally, two patients died of lung metastases, one died of brain metastasis, and one died of liver metastasis.

**Figure 1:**
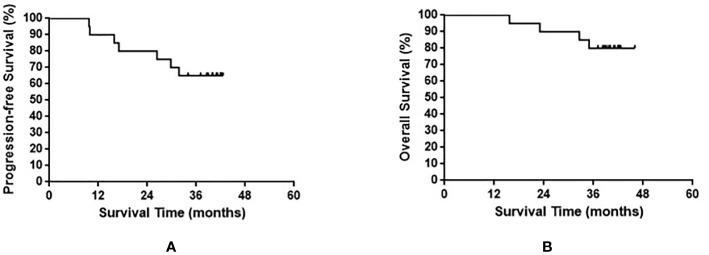
Progression-free survival **(A)** and overall survival **(B)** in all small cell lung cancer patients enrolled in the present study.

**Figure 2:**
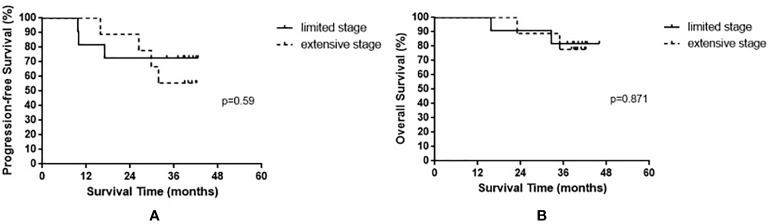
Progression-free survival **(A)** and overall survival **(B)** in small cell lung cancer patients based on limited stage and extensive stage.

The median OS of all enrolled patients was not reached, and the median progression-free survival (PFS) was 38.8 months (range, 9.7–42.6 months). The results are shown in **Figure 1**. Among the nine patients with limited-stage SCLC, three experienced recurrence and two died. Among the 11 patients with extensive-stage SCLC, four experienced relapse and two died. No statistically significant differences were observed in the PFS and OS between these two groups (p = 0.590 and p = 0.871 for PFS and OS, respectively). The results are shown in **Figure 2**.

### CTC Counts Before and After PCI

CTCs were detected in all 20 patients before PCI (100% detection rate). The median baseline count of CTCs before PCI was 12 CTCs/4 ml (range, 1–43 CTCs/4 ml). After PCI, CTCs were not detectable in four patients. The median post-PCI CTC count was 4 CTCs/4 ml (range, 0–34 CTCs/4 ml).

Logistic regression analysis showed the pre-PCI CTC count was not statistically significantly correlated with gender (p = 0.974), age (p = 0.795), VALSG stage (p = 0.126), AJCC (7^th^ edition) stage (p = 0.297), T stage (p = 0.404), N stage (p = 0.307), M stage (p = 0.985), or chemotherapy efficacy (p = 0.407), and mild significantly correlated with smoking history (p = 0.041).

### Prognosis According to Pre- and Post-PCI CTC Counts

**Figure 3:**
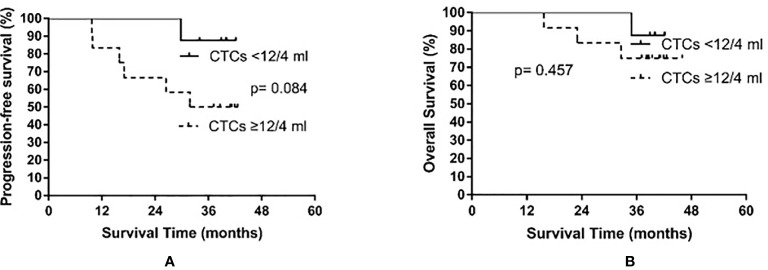
Progression-free survival **(A)** and overall survival **(B)** in small cell lung cancer patients according to circulating tumor cell (CTC) count before prophylactic cranial irradiation.

Based on the median baseline CTC count prior to PCI, patients were divided into a high baseline group (≥12 CTCs/4 ml) and a low baseline group (<12 CTCs/4 ml). The high baseline group contained 12 patients, among whom six experienced recurrence (times to relapse of 9.7, 9.9, 15.9, 17.0, 26.4, and 31.8 months) and three died. In the low baseline group comprising eight patients, only one experienced relapse (at 29.8 months after treatment) and died. The median PFS of the high baseline group was 31.8 months, while the median PFS was not reached in the low baseline group (p = 0.084). The median OS was not yet reached in either the high or the low baseline group (p = 0.457). The results are shown in **Figure 3**.

**Figure 4:**
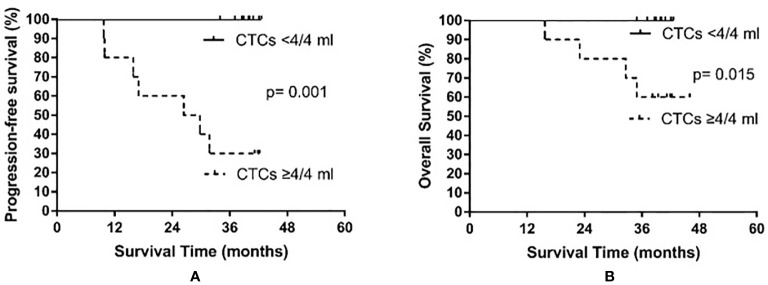
Progression-free survival **(A)** and overall survival **(B)** in small cell lung cancer patients according to circulating tumor cell (CTC) count after prophylactic cranial irradiation.

Based on the median CTC count after PCI, patients were divided into a high post-PCI level group (≥4 CTCs/4 ml) and a low post-PCI level group (<4 CTCs/4 ml). The high post-PCI level group consisted of 10 patients, among whom seven experienced recurrence and four died. The low post-PCI level group also consisted of 10 patients; however, no relapse or death occurred in the low post-PCI level group. The median PFS of the high post-PCI level group was 28.1 months, whereas the median PFS was not reached in the low post-PCI level group (p = 0.001). The median OS was not yet reached in either the high or the low post-PCI level group (p = 0.015). The results are shown in **Figure 4**.

### Relationship between Rate of *CTC* Decline and Prognosis

**Figure 5:**
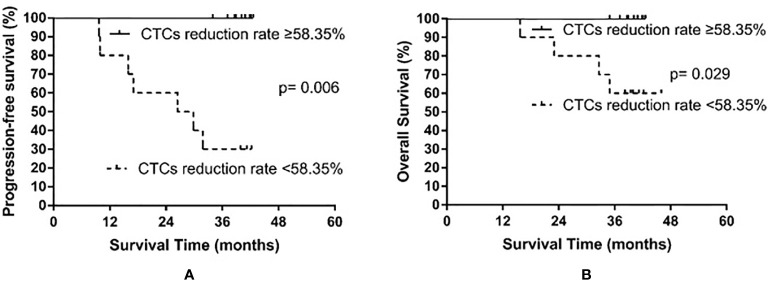
Progression-free survival **(A)** and overall survival **(B)** in small cell lung cancer patients according to the rate of circulating tumor cell (CTC) decline after prophylactic cranial irradiation.

In all patients examined, the median rate of CTC decline was 58.35% (range, −20% to 100%). Only one patient exhibited an increased number of CTCs after PCI. The patients were then divided into the high decline rate group (CTC number declined by at least 58.35%) and the low decline rate group (CTC number declined less than 58.35%). The low decline rate group consisted of 10 patients, among whom seven experienced recurrence and four died. The high decline rate group also consisted of 10 patients; however, no relapse or death occurred in the high decline rate group. The median PFS of the low decline rate group was 26.4 months, whereas the median PFS was not reached in the high decline rate group (p = 0.006). The median OS was not yet reached in either the high or the low decline rate group (p = 0.029). The results are shown in **Figure 5**.

## Discussion

A previous meta-analysis reported CTC counts are related to the prognosis of SCLC (23); however, their prognostic ability in patients who have undergone PCI was unclear. In this preliminary study, we found low post-PCI CTC counts (<4 CTCs/4 ml), not pre-PCI CTC counts, and high rates of CTC decline (≥ 58.35%) were significantly correlated with PFS and OS in SCLC patients undergoing PCI. No patients experienced treatment failure in the low post-PCI CTC level group, and OS was significantly increased compared to those in the high post-PCI CTC level group (≥4 CTCs/4 ml). Similarly, treatment failure and death rates were markedly reduced among patients who exhibited a CTC decline rate of ≥58.35% after PCI compared with those with a CTC decline rate of <58.35%. Therefore, CTC counts after PCI and the rate of CTC decline after PCI could be used to guide individualized post-PCI follow-ups and treatments for SCLC patients.

The oHSV1-hTERT-GFP technique employed in the present study has previously been used for the detection of CTCs in a variety of malignant tumors and has a number of advantages over the other available methods (19). CellSearch, which detects viable CTCs based on epithelial cellular adhesion molecule (EpCAM) expression, cannot detect CTCs with downregulated or deleted EpCAM. In addition, CellSearch cannot separate living cells for subsequent genetic testing and guidance in clinical decision making. The oHSV1-hTERT-GFP technique for CTC detection is based on telomerase-specific, replication-selective oncolytic HSV-1 that targets telomerase reverse transcriptase-positive cancer cells and expresses a green fluorescent protein that identifies CTCs (oHSV1-hTERT-GFP), with high sensitivity and specificity. This method is independent of specific molecula structures on the tumor cell surface and can be used to isolate intact living cells for further analysis of tumor cell characteristics and molecular subtypes (21). For example, compared with the CellSearch technique, the oHSV1-hTERT-GFP method allows for a higher detection rate of CTCs in lung cancer (33.3% vs. 71.2%, respectively, p = 0.000), and is not affected by the pathological type or stage (21). Indeed, we achieved a CTC detection rate of 100% in SCLC patients using the oHSV1-hTERT-GFP method, which was higher than that obtained using the CellSearch approach (range, 68.6–100%) (9, 11, 12, 14, 16–18) and the reverse transcription polymerase chain reaction (RT-PCR) method (range, 83.8–94.5%) (10, 15). In our previous study, all patients determined as CTC-positive by the CellSearch method were also determined as CTC-positive by the oHSV1-hTERT-GFP method (22/22, 100%). In contrast, of the 47 patients determined as CTC-positive using the oHSV1- hTERT- GFP method, only 22 patients (46.8%) were subsequently determined as CTC-positive using the CellSearch method (21). Furthermore, although the recently reported adenovirus-based technique (OBP-401) achieved a high CTC detection rate of 96%, this technique is limited as it only detects GFP-positive CTCs of a certain size (13). Therefore, the oHSV1-hTERT-GFP method is a highly sensitive and accurate method of determining the CTC counts in SCLC.

In this study, we found the time to relapse was considerably shorter in the high baseline CTC level group (i.e., ≥12 CTCs/4 ml after chemotherapy and thoracic radiotherapy but before PCI) than in the low baseline group (<12 CTCs/4 ml), although the difference was not statistically significant. Similarly, previous studies have found prognosis was related to CTC count after chemotherapy and thoracic radiotherapy. For example, Hiltermann et al. employed the CellSearch method to examine the number of CTCs in 59 SCLC patients before treatments, after one cycle of chemotherapy, and after combined chemotherapy and thoracic radiotherapy but before PCI (17). In their univariate analysis, PFS and OS were significantly improved in patients with a pre-PCI CTC count of <2 compared with those with a pre-PCI CTC count of ≥2 (PFS, 7.9 months vs. 3.9 months, p = 0.007; OS, 12.3 months vs. 8.1 months, p = 0.05) (17). Naito et al. also used the CellSearch technique to determine the number of CTCs in 51 patients with SCLC before treatment and after chemotherapy combined with thoracic radiotherapy but before PCI (18). They found OS was significantly prolonged in patients with a pre-PCI CTC count of <8 compared with that in patients with a pre-PCI CTC count of ≥8 (OS, 13.9 months vs. 4.1 months, p = 0.0096) (18). Similar results were obtained in the present study, which suggests the pre-PCI CTC level after combined chemotherapy and thoracic radiotherapy might indicate the recurrence of SCLC and the survival of patients. Indeed, we found patients with a high baseline CTC count experienced systemic progression soon after PCI, and two patients developed brain metastasis. Therefore, whether intensive systemic therapy rather than PCI should be applied to patients with a high baseline CTC count is worthy of further investigation.

We also found recurrence and survival were significantly worse in patients with a post-PCI CTC count of ≥4 CTCs/4 ml compared with patients with a post-PCI CTC count of <4 CTCs/4 ml. All patients who experienced recurrence or died were in the high post-PCI CTC level group. In addition, recurrence and survival were significantly worse in patients who exhibited a CTC decline rate of <58.35% following PCI than in those with a CTC decline rate of ≥58.35%, and all patients who experienced recurrence or died belonged to the low decline rate group. As local therapy, PCI which mainly aim at the micro-metastases of the brain, theoretically should not affect micro-metastases from other tissues. We cannot provide any explanation for the observed reduction in CTC counts and need further investigation. In addition, the continuous decrease of CTC count indicates that the impact of chemotherapy and thoracic radiotherapy is continuously effective, and theoretically, the prognosis of patients should be better. Indeed, all patients, except one, exhibited some degree of CTC decline, which to some extent, indicates the effectiveness of PCI treatment or the delayed effect of combined chemotherapy and thoracic radiotherapy.

Our results also indicate that patients who experience a significant decline in CTC number benefit more from anti-cancer treatment. Consistently, others have reported CTC decline after treatment in patients with SCLC is indicative of good PFS and OS. For example, Huang et al. used the CellSearch technique to determine the pre- and post-chemotherapy CTC count in 25 patients with extensive-stage SCLC and found the median rate of CTC decline was 98.7% (12). They reported the rate of CTC decline correlated with OS (p = 0.09), and for every 1% decrease in the CTC decline rate, the risk of death increased by 2.8% (12). In another study, Normanno et al. reported a median rate of CTC decline of 86.5% after one cycle of chemotherapy in 60 patients with extensive-stage SCLC using the CellSearch technique (14). They also found the higher the CTC decline, the lower the risk of death (hazard ratio [HR] 0.24, 95% confidence interval [CI] 0.09–0.61) (14). Furthermore, the OS significantly improved in patients with a CTC decline rate of ≥89% compared with that in patients with a CTC decline rate of <89% (OS, 7.2 months vs. 4.2 months) (14). Therefore, our results and those of others suggest patients with a high post-PCI CTC count, including those that exhibit a low rate of CTC decline, have a very high risk of treatment failure and death. For these patients, it is necessary to emphasize systemic treatment to improve therapeutic efficacy. It is noteworthy that a good prognosis and disease control were achieved in patients with a high CTC count before PCI but who also exhibited a rapid decline in CTC number after PCI; however, due to the limited number of patients included in the analysis, such findings require further verification.

The present study has some limitations. First, only a small number of patients were included in the preliminary study, rendering it difficult to draw a definitive conclusion. Second, the present study included patients with limited-stage SCLC and patients with extensive-stage SCLC, which causes a certain bias despite the lack of significant differences in relapse and survival between the two groups. Third, the decline in CTC count after PCI may be related to previous chemotherapy and thoracic radiotherapy, and the present study failed to determine CTC numbers in the patients during the initial treatment and the follow-up period. Therefore, the relationship between the dynamic changes in tumor load and CTC numbers could not be accurately reflected.

## Conclusions

Our preliminary results show the post-PCI CTC count and the rate of CTC decline after PCI could predict recurrence and survival in patients with SCLC. Therefore, these CTC counts can be used to guide individualized post-PCI follow-ups and treatments for SCLC patients. A trial including further expansion of the sample size and multiple-time-point monitoring has been registered to verify this finding (ChiCTR1900024021).

## Data Availability Statement

The raw data supporting the conclusions of this article will be made available by the authors, without undue reservation.

## Ethics Statement

The studies involving human participants were reviewed and approved by the Ethics Committee, National Cancer Center/National Clinical Research Center for Cancer/Cancer Hospital, Chinese Academy of Medical Sciences and Peking Union Medical College (approval no. NCC2015 G -77). The patients/participants provided their written informed consent to participate in this study. Written informed consent was obtained from the individual(s) for the publication of any potentially identifiable images or data included in this article.

## Author Contributions

LD and YZ contributed to the research design, case collection, data analysis, and manuscript writing. WZ, LF, and KZ performed the CTC detection assay. WW and ZZ participated in the case collection, sample quality control tests, and data analysis. LW and ZH participated in the research design, data analysis and manuscript writing. All authors contributed to the article and approved the submitted version.

## Funding

This work was supported by the National Natural Science Foundation of China (81572971), the Basic Scientific Research Funding Program of the Chinese Academy of Medical Sciences (2016ZX310012), the Beijing Hope Marathon Special Fund (LC2016L03), and the National Key R&D Program of China (2017YFC1311000).

## Conflict of Interest

The authors declare that the research was conducted in the absence of any commercial or financial relationships that could be construed as a potential conflict of interest.
